# Engineering of TurboID-Wingless for the identification of Wingless interactors through *in vivo* proximity labelling

**DOI:** 10.17912/micropub.biology.001210

**Published:** 2024-05-29

**Authors:** Ana-Miruna Androniciuc, Edward W. Tate, Jean-Paul Vincent

**Affiliations:** 1 The Francis Crick Institute, London, England, United Kingdom; 2 Department of Chemistry, Imperial College London, London, England, United Kingdom

## Abstract

Wnt signalling coordinates growth and cell fate decisions during development and mis-regulation of Wnt signalling in adults is associated with a range of conditions, including cancer and neurodegenerative diseases. Therefore, means of modulating Wnt proteins and/or cofactors could have significant therapeutic potential. As a first step towards enumerating the Wnt interactome, we devised an *in vivo* proximity labelling strategy to identify proteins that interact with Wingless (Wg), the main *Drosophila* Wnt. We engineered the *wingless* locus to express a functional TurboID-Wg fusion at endogenous levels and identified *in vivo* interactors by streptavidin pull-down from embryos, followed by mass spectrometry. Further analysis may in future extend the screen coverage and deliver functional validation of the newly identified interactors.

**Figure 1. Development of TurboID-Wg as a tool for the unbiased identification of Wg interactors through in vivo proximity labelling:**
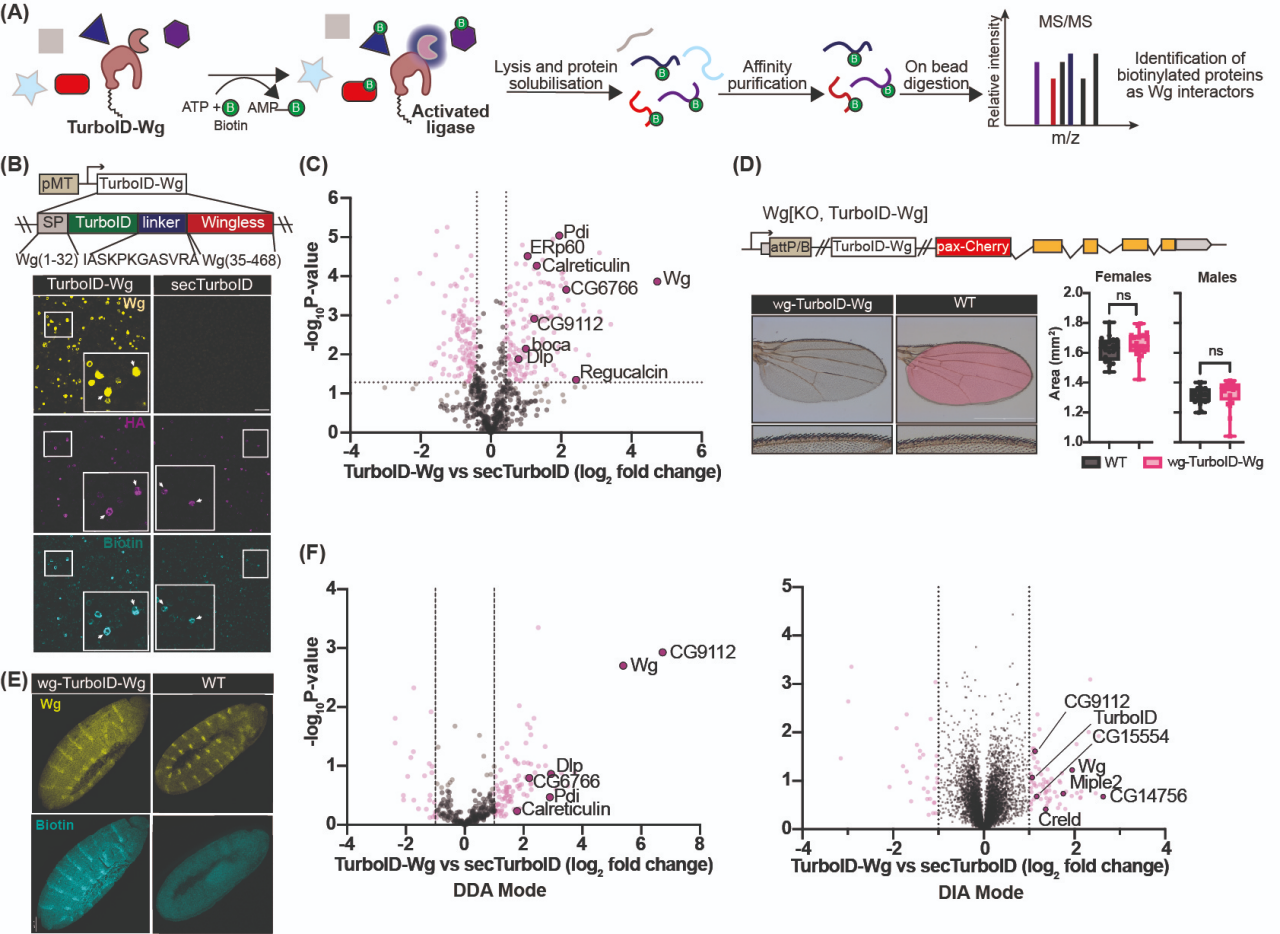
**(A) Schematic workflow for proximity labelling and identification of Wg interactors.** In the presence of ATP and biotin the TurboID-Wg fusion protein can biotinylate nearby proteins in vivo. After lysis, these proteins were isolated by affinity purification and subsequently identified by mass spectrometry. **(B) Expression of TurboID in S2 cells leads to an overall increase in biotinylated proteins.** Top: Map of TurboID-Wg expressing plasmid transfected in S2 cells; pMT refers to the metallothionein promoter. Bottom: Immunofluorescence staining of S2 cells transfected with Dlp-HA along with either TurboID-Wg (left) or secTurboID (right) and stained with anti-Wg (yellow), anti-HA (magenta) and streptavidin (cyan). The insets show at high magnification examples of successfully co-transfected cells, marked by white arrows. Scale bar is 50 μm. Representative images of n = 2 independent experiments. **(C) Proteomic analysis of TurboID-Wg vs sec-TurboID expressing S2 cells.** Volcano plot comparing the biotin enriched proteome of S2 cells transfected with Dlp-HA and either TurboID-Wg (right) or secTurboID (left). Proteins of interest are coloured purple. Significant proteins are coloured pink (Calculated by t-test; S0 0.2; FDR 5%). Vertical (x = 0.5 and x = -0.5) and horizontal (y = 1.3) intercepts are used to approximately delineate the quadrant of proteins significantly enriched. n = 3 (biological), n = 1 (technical). **(D) Expression of TurboID-Wg from the endogenous *wingless* promoter ( *wg-TurboID-Wg* ) fully recapitulates Wg function.** Top: schematic of modified *wingless* genomic locus expressing TurboID-Wg. Bottom: Whole wing and magnified wing margins of *wg-TurboID-Wg* (left) and WT (right), with the *wg-TurboID-Wg* wing overlaid in pink shading. Quantification of wing surface area from WT (black, n = 20 females and 23 males), wg-TurboID-Wg (pink, n = 23 females and 24 males). Statistical significance was assessed using an unpaired t-test. **(E) Protein biotinylation is specifically enhanced in the Wg-expressing domain of *wg-TurboID-Wg* embryos.** Immunofluorescence staining of embryos expressing WT Wg (right) or TurboID-Wg (left) stained with anti-Wg (yellow) and streptavidin (cyan). Scale bar is 50 μm. Representative images from n = 4 independent experiments. **(F) Proteomic data sets from *wg-TurboID-Wg* embryos.** Volcano plots comparing the biotin-enriched proteome of embryos expressing TurboID-Wg (right) or secTurboID (left) determined by Student t-test. Vertical (x = 1.0 and x = -1.0) intercepts are used to delineate enriched proteins. Above threshold proteins are coloured pink and proteins of interest are coloured purple. Mass spectrometry acquisition was performed in data-dependent mode (left) or data-independent mode (right). For DDA: n = 3 (biological). n = 1 (technical); for DIA: n = 3 (biological). n = 4 (technical). A full list of proteins identified in all mass spectrometry experiments can be found in the extended data.

## Description

Four decades of Wnt signalling research have led to an appreciation of this pathway’s molecular sophistication. Many components of this pathway have been identified and characterised through genetic (Bartscherer et al., 2006; Proffitt and Virshup 2012; Bhanot et al., 1996) and structural (Janda et al., 2012; McGough et al., 2020; Nygaard et al., 2021; Liu et al., 2022) approaches. Nevertheless, some questions remain, including the mechanisms of dissociation of Wnt from its chaperone Wls in producing cells, Wnt extracellular transport (McGough et al., 2020) and Wnt handover to Frizzled (Fzd) receptors. Work in *Drosophila melanogaster* has been instrumental to Wnt research owing to its powerful genetics and the high degree of evolutionary conservation of Wnt pathway components. *Drosophila* is a particularly versatile model for expression of modified variants at endogenous level in a complex tissue context. TurboID (Branon et al., 2018) is an engineered biotin ligase that catalyses the biotinylation of proximal proteins with one, or more, biotin molecules, provided that they have surface-exposed lysine residues and biotin and ATP are readily available. Biotin tagging of the interactors in turn allows their separation from the complex protein mixture and subsequent identification through mass spectrometry ( Figure 1 . (A)). We chose TurboID because of the relatively low toxicity of labelling protocols compared, for example, to APEX2 (Lam et al., 2015) and fast kinetics compared to legacy ligases such as BioID (Roux et al., 2012) , which together enable the capture of transient interactions *in vivo* .

We based our design of *TurboID-Wg* on the previously published *eGFP-Wg* strain (Port et al., 2014) which is morphologically normal in homozygotes, indicating that *eGFP-Wg* is fully functional ( Figure 1 . (B), top panel). To weed out non-specific interactors in the secretory pathway, we designed a form of TurboID ( *secTurboID* ) that contains the Wg signal peptide but lacks the rest of the protein. To confirm that these fusion proteins induce the biotinylation of nearby proteins, we introduced the respective coding sequences in an inducible pMT vector, which was then transiently transfected in *Drosophila* Schneider’s (S2) cells. The cells were co-transfected with a plasmid expressing Dlp, a known Wg interactor (McGough et al., 2020) . Immunofluorescent staining of the S2 cells confirmed both TurboID-Wg and secTurboID induced biotinylation in the transfected cells ( Figure 1 . (B), right panel). We then compared the biotin-enriched proteome of cells expressing TurboID-Wg or secTurboID. As expected, both Wg and Dlp were specifically enriched in the biotinylated fraction of TurboID-Wg expressing cells ( Figure 1 . (C). Various ER-resident proteins and chaperons, including Pdi, ERp60, Regucalcin and Boca ( Figure 1 . (C)), which have previously been shown to play a role in the Wnt pathway (Li et al., 2022; Kondylis et al., 2011) were also enriched despite not being overexpressed. Of note, Calreticulin which was recently shown to play a direct role in Wnt secretion (Qi et al., 2023) was also specifically biotinylated by TurboID-Wg but not by secTurboID. These results suggest that functionally relevant Wnt-interacting proteins can be identified by proximity labelling.

Our cell-based approach also identified two previously uncharacterised secreted proteins, those encoded by CG9112 and CG6766, whose potential functions are currently only derived from computational assignment of protein domains (Paysan-lafosse et al., 2022) and gene ontology (Gaudet et al., 2011) . CG9112 contains a calcium binding EF-hand domain (Paysan-lafosse et al., 2022) and is the orthologue of human Reticulocalbin2 (RCN2) (Gaudet et al., 2011) , therefore it is predicted to have calcium binding properties. CG6766 is the orthologue of human endoplasmic reticulum lectin 1 (ERLEC1) (Gaudet et al., 2011) and is predicted to contain a mannose-6-phosphate receptor binding domain (Paysan-lafosse et al., 2022) and to be involved in the ubiquitin dependent ERAD pathway and the ER unfolded protein response (Gaudet et al., 2011) . Both CG9112 and CG6766 are ubiquitously expressed in the embryo (Tomancak et al., 2002, 2007; Hammonds et al., 2013) and the wing imaginal disc (Deng et al., 2019) which would allow them to interact with Wg, however this remains to be determined experimentally.

To improve the physiological relevance of our screen, we next proceeded to generate animals expressing TurboID-Wg from the endogenous *wingless* locus, using an AttP landing site that knocks out endogenous gene function (Baena-Lopez et al., 2013) . The resulting animals, which we refer to as *wg-TurboID-Wg* , were found to be normal, even as homozygotes. This included classic indicators of normal Wg signalling such as wing size (Giraldez and Cohen 2003) and patterning of the wing margin (Couso, Bishop, and Arias 1994) ( Figure 1 . (D)). To assess the functionality of the TurboID portion of the fusion protein, embryos were collected from females raised with food supplemented with biotin (see Methods) and stained with streptavidin. As can be seen in Figure 1 . (E), biotinylated proteins are readily detected in and around the domain of Wg expression (shown with anti-Wg). No such pattern of biotinylation was seen in similarly treated WT embryos. Next, we generated a strain expressing secreted TurboID from the endogenous *wingless* locus (same insertion site as that used for *wg-TurboID-Wg* ). Homozygous embryos displayed the hallmark phenotype of null *wingless* mutants, as expected since they produce no functional Wg. However, biotinylation was detected in the Wg expression domain of heterozygotes, indicating that TurboID is functional and suggesting that this strain can be used as a baseline control for the identification of specific Wg interactors.

We therefore proceeded to compare the biotin-enriched proteome of embryos expressing TurboID-Wg and secTurboID, using the same data-dependent acquisition (DDA) method used to generate the S2 proteomics data. By comparison to the cell-based data, the *in vivo* data suffered from generally low numbers of identifiable proteins and poor reproducibility between biological replicates. This is likely due to the complexity of embryo lysate samples, as previously reported in other proteomics studies of embryos (Tan et al., 2009; Taraszka et al., 2005) . Under the circumstances, we chose to consider hits that show a log _2_ fold change > 1 as enriched, even though they were not necessarily t-test statistically significant. Reassuringly, Wg was found among the proteins enriched in *wg-TurboID-Wg* embryos (relative to *wg-secTurboID* embryos). In addition, CG9112 and CG6766, which were previously identified in the cell-based proteomics data were also found in the enriched fraction, together with Dlp, Pdi and calreticulin ( Figure 1 .(F), left panel). However, several key Wg interactors such as Porcn, Wls, Fz2, Arrow or Notum were not identified, possibly because of poor protein solubility (Porcn, Wls, Fz2), a small number of exposed lysine residues (Porcn), or possibly due to an adverse effect of the extracellular environment on biotinylation efficiency (Fz2, Arrow, Notum).

In an attempt to improve the sensitivity of the detection, we assessed whether a recently developed data-independent acquisition (DIA) method (Meier et al., 2020) would improve the range of identified proteins. In DIA mode, many more proteins could indeed be identified (6000 compared to 700 in DDA mode), with Wg remaining one of the top enriched proteins ( Figure 1 . (F), right panel). All the known Wg interactors (except Porcn) were detectable but equally so in the *wg-TurboID-Wg* and *wg-secTurboID* datasets. Reproducibility from one biological repeat to another remained an issue. Although encouraging, these results suggest that improvements in the lysis and protein solubilisation protocol will be needed to increase data reproducibility. We suggest that, once this is achieved, the approach we have initiated could be used to identify proteins that interact with Wg at various developmental stages, or in tissue-specific contexts, and thus help build a complete Wg interactome.

## Methods

**Drosophila husbandry and fly genetics:** Fly strains were raised on standard fly food at 25°C. DNA injections to generate transgenic fly lines were performed by the Crick Fly Facility. The fly lines used in this study are summarised in the Reagents Table. w1118 flies were used as WT stock. Standard fly handling techniques were employed (Roberts 1998) . Balancer chromosomes and markers used have been described previously (Lindsley and Zimm 1992) .

**Immunostaining:** Cells : Drosophila S2 cells were cultured at 25˚C in Schneider’s medium + L-glutamine containing 10% (v/v) fetal bovine serum (FBS) and 0.1 mg/mL Pen/Strep. Cells were plated in 10 cm dishes and co-transfected with 1.5 μg of each plasmid (tubulin-Dlp-HA and either pMT-TurboID-Wg and pMT-secTurboID) using the Effectene Transfection Reagent Protocol. After 24h, CuSO _4_ to a final concentration of 500 μM was added to each dish to induce protein expression. Protein expression was tested by immunofluorescence or Western blot after another 48 h.

Embryos : Fly cages were set up overnight and embryos were collected on grape juice plates supplemented with fresh yeast. Embryos were bleached in 100 % sodium hypochlorite solution on Whatman filter paper for 2-3 minutes for dechorination. The embryos were then rinsed 3 times with PBS and transferred to a glass vial containing 37% formaldehyde in heptane. Embryos were fixed for 90 minutes after which they were transferred to a plastic dish using a Pasteur pipette. The heptane was allowed to evaporate before adding PBS. The vitelline membrane was removed manually using a sharp needle. Triton X-100 was added to a final concentration of 0.1-0.3% and the embryos were transferred to an Eppendorf tube and permeabilised for 10 minutes before blocking for 1h in PBT + 0.2% NDS. Embryos were incubated in blocking solution containing primary antibodies overnight. After 3 PBT washes, embryos were incubated with secondary antibodies and Hoechst for 2 hours at room temperature before mounting in Aqua-Mount. The primary antibodies used were as follows: mouse anti-Wingless (1:500), Streptavidin-AlexaFluor555 (1:500), Streptavidin-AlexaFluor488 (1:500), rabbit anti-HA (1:500). The secondary antibodies used were as follows: goat anti-mouse Alexa488 plus (1:500), goat anti-mouse Alexa555 (1:500), goat anti-rabbit Alexa555 (1:500), goat anti-rabbit Alexa488 (1:500).

**Adult wing imaging and surface area quantification:** Adult flies were stored in 100% isopropanol prior to dissection. Flies were washed once more in 100% isopropanol after which the adult wings dissected in 100% isopropanol and mounted with Euparal. Images were acquired on a wide field microscope Zeiss Axiovert 200M. Wing blade area was calculated using an in-house MATLAB script written by Dr. Anqi Huang.

**Mass spectrometry:** Cells : Cell lysates were obtained by incubating cells in 1x RIPA for a few minutes on ice then spun at 4°C for 15 minutes twice to remove cell debris. Proteins were precipitated by the sequential addition of 0.5x sample volume of H _2_ O, 1x sample volume of MeOH and 0.25x sample volume of CHCl _3_ followed by vortexing and centrifugation at 6000 rpm for 2 minutes. The top layer, made up of water and chloroform, was removed and a further 500 μL of MeOH were added and the protein pellet disrupted by sonication. Samples were then microfuged at maximum speed for 5 minutes. This was repeated twice, after which the methanol was removed and the pellet allowed to dry before resuspending in 1/10 ^th^ of the initial volume of 2% SDS in 50 mM HEPES pH 8. Further 50 mM HEPES pH 8 was added to a final concentration of 0.2% SDS. Protein concentration was calculated using Pierce BSA Assay and 1.8 mg of total protein were used for the proteomics experiment.

The neutravidin-coated agarose beads were washed 5x with 100 mM triethylammonium bicarbonate (TEAB), pH 8.0, before being treated with 5x volume of 100 mM TEAB, pH 8.0, containing 0.2% formaldehyde and 25 mM NaBH _3_ CN. The mixture was incubated for 1h at room temperature with gentle rotation. Beads were washed 2x with 100 mM TEAB, pH 8.0, containing 1% ethanolamine to quench the reaction. The beads were washed 3x with 50 mM HEPES, pH = 8.0 and stored at 4°C until needed. 25 μL of beads is sufficient to pull down 1 mg of total protein.

Biotinylated proteins were pulled-down overnight at 4°C, before they were recovered, the supernatant removed (and discarded) and washed with 0.2% SDS in 50 mM HEPES, pH 8.0 (x2) followed by 50 mM HEPES, pH 8.0 (x4). The beads were recovered by centrifugation at 5000 rpm. After the final washing step, the beads were resuspended in 100 μL of 50 mM HEPES, pH 8.0 and incubated with LysC (20 μg/100μL) for 1h. The supernatant was isolated and transferred to a LoBind Eppendorf tube. 5 mM TCEP and 10 mM CAA were added and incubated for 10 minutes with gentle shaking. Trypsin was added (20 μg/100 μL) in Tris 50 mM pH 8.3 and incubated at 37°C with shaking overnight. TFA was added to a final concentration of 0.5%, and the samples filtered through a 0.22 μM membrane before submission to the Crick Proteomics science technology platform.

Peptides were analysed either by nano-scale capillary LC-MS/MS using an Ultimate 3000 nanoRSLC HPLC (Thermo Scientific) whereby 1–10 μl of acidified protein digest was loaded onto a 20 mm x 75 μm Pepmap C18 trap column (Thermo Scientific) prior to elution via a 50 cm x 75 μm EasySpray C18 column into a Lumos Tribrid Orbitrap mass spectrometer (Thermo Scientific). A 90 min binary gradient of 6%-40% buffer B (80% ACN, 0.1% formic acid) over 63 min was used prior to washing and re-equilibration (buffer A, 2% ACN, 0.1% formic acid; buffer B, 80% ACN, 0.1% formic acid), or by using an Evosep One LC system (EvoSep Biosystems) directly coupled to an Orbitrap Fusion Lumos tribrid mass spectrometer (Thermo Scientific). Reverse phase separations were performed at a flow rate of 500 nL/min on an EV1064 ENDURANCE analytical column (100 μm × 8 cm, 3.0 μm particle size; Evosep Biosystems) using the vendor’s predefined 30 samples per day gradient method. The Orbitrap was operated in ‘TopS’ Data Dependent Acquisition mode with precursor ion spectra acquired at 120k resolution in the Orbitrap detector and MS/MS spectra at 32% HCD collision energy in in the ion trap. Automatic Gain Control was set to Auto for MS1 and MS2. Maximum injection times were set to ‘Standard’ (MS1) and ‘Dynamic’ (MS2). Dynamic exclusion was set to 20 s.

Peptide search was performed in MaxQuant (Cox and Mann 2008) , with Oxidation (M) and acetyl (Protein N-term) set as variable modifications, while Carbamidomethyl (C) was set as fixed. The modification of lysine residues with a mass corresponding to biotin was also included. Instrument type was Orbitrap, with its predefined settings. Tripsyn/P was selected as the digestion mode with a maximum of 2 missed cleavages. Label-free quantification was selected, with the LFQ minimum ratio count set to 2. FASTA file corresponding to the *Drosophila* proteome, with Wg modified to TurboID-Wg was used. Data analysis was performed in Perseus (Tyanova et al., 2016) . LFQ intensity values were loaded into the matrix and data filtration was done by removing rows based on ‘potential contaminant’, ‘reverse’ and ‘only identified by site’. Data was then log _2_ transformed and filtered based on valid values in the TurboID-Wg condition. Data was normalised by subtraction of median and missing values were imputed before generating volcano plots using a pairwise Student’s t-test.

Embryos : To generate embryo lysates, flies were placed on fly food enriched with biotin to a final concentration of 500 μM and following a full fly life cycle of biotin feeding (approx. 2 weeks), freshly emerged adults (approx. 300 flies/genotype) were placed in a 1L cage covered with a 10 cm grape juice plate. After 2-day acclimatisation, flies were allowed to lay embryos on a fresh plate overnight (~22h). Embryos were collected in mesh baskets and washed thoroughly. They were then bleached with 50% household bleach solution for 2-3 minutes and then washed again before being transferred to Eppendorf vials containing cold lysis buffer (1x RIPA buffer + 0.5% NP-40 with protease inhibitors). Approximately equal volumes of embryos and buffer were used (~500 μl each). Embryos were homogenised either using a pestle and an electric homogeniser, or by sonication with a BioRaptor instrument, and placed on ice. The homogenate was microfuged at max speed for 20 mins at 4°C. The clear lysate and the lipid layer were transferred to a fresh Eppendorf, and microfuged at max speed for 15 min at 4°C twice, and stored at -80°C.

**DDA mode** : Proteins were precipitated using the MeOH/ CHCl _3_ protocol and after resuspension, 10 mg of total protein were used for the pull-down. The rest of the protocol is as described in the cellular proteomics experiment.

**DIA mode** : Proteins were precipitated by the addition of 4x sample volume of ice-cold 100% acetone followed by rotation overnight at 4°C. Samples were spun at maximum speed for 30 minutes at 4°C, the supernatant removed and the protein pellet washed twice with ice-cold 80% acetone/20% water with 10-minute centrifugation steps in between. The supernatant was removed and the pellet allowed to dry before resuspending in buffer by sonicating for 15 minutes. 10 mg of total protein were used for the pull-down. The neutravidin coated magnetic beads were washed three times with PBS and aliquoted. 400 μL of beads were incubated with 360 μL of PBS and 40 μL of 200 mM Sulfo-NHS-Acetate (SNA) for 15 minutes at room temperature with gentle rotation. Beads were recovered using a magnetic stand and the supernatant removed. A further 360 μL of PBS and 40 μL of 200 mM SNA were added to the beads and incubated for 30 minutes. To quench any unreacted SNA, 80 μL of Tris, pH 7.5 was added to the mixture after which the beads were recovered and washed 3x with PBS and stored at 4°C until needed. Biotinylated proteins were immunoprecipitated overnight at 4°C. Beads were recovered using a magnetic stand, the supernatant was removed (and discarded) and the beads were washed. On-bead digestion, reduction and alkylation were performed as described in the cellular proteomics experiment. TFA was added to a final concentration of 0.5%, and the samples filtered through a 0.22 μM membrane before submission to the Crick Proteomics science technology platform.

Peptides were analysed using an Evosep One LC system (EvoSep) coupled to a timsTOF Pro 2 mass spectrometer (Bruker Daltonik GmbH) using a commercial 150-mm analytical column (EV1113 ENDURANCE COLUMN, Evosep Biosystems) and an integrated Captive Spray Emitter (IonOpticks). Buffer A was 0.1% formic acid in water, Buffer B was 0.1% formic acid in acetonitrile. Data was collected using diaPASEF with 1 MS frame and 9 diaPASEF frames per cycle with an accumulation and ramp time of 100 ms, for a total cycle time of 1.07 seconds. The diaPASEF frames were separated into 3 ion mobility windows, in total covering the 400 – 1000 m/z mass range with 25 m/z-wide windows between an ion mobility range of 0.64–1.4 Vs/cm ^2^ . The collision energy was ramped linearly over the ion mobility range, with 20 eV applied at 0.6 Vs/cm ^2^ to 59 eV at 1.6 Vs/cm ^2^ .

The peptide search was performed in DiaNN, using diaPASEF.d (Meier et al., 2020) mode. Samples were first run in library-free mode, FASTA digest and deep learning-based spectra, RTs and IMs predictions were selected. The protease selected was Tripsyn/P with a maximum of 1 missed cleavages. N-term M excisions, C carbamidomethylation, Ox (M) and Ac (N-term) were set as modifications, with the maximum number of variable modifications set to 2. The use of isotopologues was selected, as well as match between runs and no shared spectra. Precursor FDR was set to 1%. Protein inference was set to Genes, Neural network classifier to Single-pass mode, Quantification strategy to Robust LC (high precision), Cross-run normalisation to RT-dependent, Library generation to Smart profiling and Speed and RAM usage to optimal results. FASTA files corresponding to the *Drosophila* proteome, together with files for TurboID-Wg and secTurboID were used.

**Extended data:** Full list of proteins identified in the three proteomics experiments described above. The mass spectrometry proteomics data have been deposited to the ProteomeXchange Consortium via the PRIDE (Perez-riverol et al., 2022) partner repository with the dataset identifier PXD051416.

## Reagents

**Table:** 

**Reagent**	**Source**
Drosophila S2 cells	Drosophila Genomics Resource Centre
Schneider’s medium + L-glutamine	Sigma
Fetal bovine serum	Life Technologies
Pen/Strep	Life Technologies
Effectene Transfection Reagent	Qiagen
Mouse anti-Wingless	DSHB 4D4
Streptavidin-AlexaFluor555	ThermoFisher, S21381
Streptavidin-AlexaFluor488	ThermoFisher, S11223
Rabbit anti-HA	CST, 3724S
Goat anti-mouse Alexa488 plus	ThermoFisher, A32723
Goat anti-mouse Alexa555	ThermoFisher, A32727
Goat anti-rabbit Alexa555	ThermoFisher A32732
Goat anti-rabbit Alexa488	ThermoFisher, A32731
1x RIPA buffer	Millipore, 20-188
Pierce BSA Assay	ThermoFisher
Neutravidin coated agarose beads	ThermoFisher, 29200
Neutravidin coated magnetic beads	Cytiva, 1528692
Sulfo-NHS-Acetate	ThermoFisher, 26777
	
**Fly strain genotype**	
*w1118*	Lab stock
*WgKOattp/CyO*	Lab stock
*eGFP-Wg*	Lab stock (from Port et al., 2014)
*wg-TurboID-Wg*	This study
*wg-secTurboID/CyO*	This study

## Extended Data

Description: Archive of files containing full list of proteins identified in the proteomics experiments (associated figure in file name). Resource Type: Dataset. DOI: 10.22002/68evq-92p83
